# Impact of Lymphadenectomy on Outcomes of Early-Stage Ovarian Cancer: A Systematic Review and Meta-Analysis

**DOI:** 10.3389/fsurg.2021.682348

**Published:** 2021-05-25

**Authors:** Guorong Yao, Xiaotian Jin

**Affiliations:** ^1^Department of Obstetrics and Gynaecology, Huzhou Central Hospital, Affiliated Central Hospital Huzhou University, Huzhou, China; ^2^Gynecology Clinic, Huzhou Maternity & Child Health Care Hospital, Huzhou, China

**Keywords:** ovarian cancer, lymph node, surgery, survival, pelvic nodes, para-aortic nodes

## Abstract

**Objective:** The study aimed to assess if additional lymphadenectomy with primary staging surgery improves overall survival (OS) and disease-free survival (DFS) of early-stage ovarian cancer (ESOC).

**Methods:** PubMed and Embase databases were searched for any type of study comparing OS or DFS between lymphadenectomy and control groups for any type of ESOC. Adjusted hazard ratios (HR) were pooled in a random-effects model.

**Results:** Twelve studies were included. Meta-analysis indicated that lymphadenectomy is associated with significantly improved OS only for epithelial tumors (HR 0.75 95% CI 0.68, 0.82 I^2^ = 0% *p* < 0.00001) but not for malignant germ cell tumors (HR 1.31 95% CI 0.88, 1.94 I^2^ = 0% *p* = 0.18). Single studies indicated a tendency of improved OS with lymphadenectomy which was significant for ovarian carcinosarcoma but not for sex cord-stromal tumors. On meta-regression of all histological types, the percentage of patients with lymph node metastasis in the lymphadenectomy group was not found to influence the effect size. Meta-analysis also indicated that lymphadenectomy is associated with significantly improved DFS for epithelial tumors (HR 0.59 95% CI 0.45, 0.77 I^2^ = 0% *p* < 0.0001). Single studies on malignant germ cell and sex cord-stromal tumors failed to demonstrate any significant beneficial effect of lymphadenectomy on DFS.

**Conclusions:** Within the limitations of the review, lymphadenectomy may improve OS and DFS for epithelial ESOC. Scarce data suggest that lymphadenectomy is not associated with improved outcomes for malignant germ cell and sex cord-stromal tumors but may benefit ovarian carcinosarcoma. Large-scale RCTs and robust observational studies shall improve current evidence.

## Introduction

Ovarian cancer remains one of the most lethal gynecological malignancies worldwide. It is the second most common genital malignancy and the most common cause of genital cancer-related death in females (1). Ovarian cancer not only impacts survival but also leads to significant reduction in the overall quality of life and sexual functions in affected individuals (2). Importantly, only about 25% of these tumors are detected at an early stage with the majority of cases diagnosed with advanced disease (3). Indeed, distinguishing adnexal masses to recognize ovarian cancer has been a challenge for clinicians. Over the years, research has been directed toward the use of tumor markers like CA125, HE4, PRSS8, FOLR1, KLK6/7, GSTT1 and the use of transvaginal ultrasonography for early recognition of these tumors. Also different validated models like International Ovarian Tumor Analysis (IOTA) and the Assessment of Different NEoplasias in the AdneXa (ADNEX) model have been developed to aid in preoperative characterization of the adnexal pathology (4). Despite such advances, the survival with ovarian cancer remains low, ranging from 30 to 40% (5).

Surgical treatment remains the primary mode of management of ovarian cancer. However, as with any surgical intervention inherent complications exists (6, 7). Over the years, there has been a trend to use minimally invasive approaches for managing this disease. Laparoscopy is being increasingly used to assess the resectibility of ovarian cancer (8). The use of minimally invasive laparoscopic and robotic approaches for surgery has been suggested to improve perioperative outcomes with minimal impact on overall survival (9). A recent study by Lago et al. (10) has demonstrated improved psychological impact of minimally invasive surgery as compared to traditional laparotomy in patients with advance ovarian cancer. However, irrespective of the surgical approach, the quality and extent of the excision are known to be important factors influencing survival in these patients (11, 12).

Metastasis to the pelvic and para-aortic lymph nodes is frequently seen in case of ovarian cancer as compared to other gynecological tumors and sampling of these nodes is important for the staging of the disease (13). However, owing to the low rate of lymph node involvement in early-stage ovarian cancer (ESOC), sentinel lymph node technique has been recently suggested but is still under trial stage (10). For lymph node sampling in ESOC, a distinction should be made between sampling and complete pelvic and/or para-aortic dissection i.e., lymphadenectomy which is associated with significant surgical morbidity (14, 15). Studies indicate that around 6.1 to 29.6% of ESOC have occult lymph node metastasis (16, 17). Patients with positive nodes identified via staging lymphadenectomy are usually upgraded to an advanced stage and require adjunctive therapy for the residual disease (18). However, the recent ESMO-ESGO consensus conference for ovarian cancer has questioned the need for lymphadenectomy for all histological types of ESOC. Furthermore, it advocated that lymph node dissection for re-staging purposes can be omitted if patients' management is not affected by the nodal status (19).

Whether lymphadenectomy is beneficial for advanced as well as early-stage ovarian cancer (ESOC) has been a subject of intense research in the past two decades (15, 18, 20). To date, several meta-analyses have been published evaluating the role of lymphadenectomy for ovarian cancer (21–26). However, most of these studies have focused on advanced stage ovarian cancer and to the best of our knowledge, only three meta-analysis studies (24–26) have assessed the impact of lymphadenectomy on the outcomes of ESOC. However, these reviews were focused only on epithelial ESOC and could include only a limited number of studies in their meta-analysis. At this point, it is still unclear how does lymphadenectomy benefits patients with ESOC with different histological subtypes. Therefore, the purpose of this study was to systematically search the literature and conduct a meta-analysis to assess the impact of lymphadenectomy on the outcomes of ESOC.

## Materials and Methods

The review was conducted as per the PRISMA statement (Preferred Reporting Items for Systematic Reviews and Meta-analyses) (27). The review protocol was not registered on any of the online databases. The research questions for the review were: (1) Does lymphadenectomy improve the overall survival (OS) of patients with ESOC? (2) Does lymphadenectomy improve disease-free survival (DFS) of patients with ESOC?

### Eligibility Criteria

We included the following studies in the systematic review:

(1) Any type of study [Randomized controlled trial (RCT), prospective or retrospective] conducted on patients with histologically confirmed ESOC. (2) Patients were to undergo staging surgery with or without lymphadenectomy (pelvic, para-aortic, or both). (3) Studies were to compare OS or DFS between lymphadenectomy and control groups and reported multivariable-adjusted outcomes. No restriction was placed on the histological type of ESOC.

Exclusion criteria were as follows: (1) Studies assessing outcomes of advanced ovarian cancer. (2) Studies on a mixed population of ovarian cancer and not reporting separate data for ESOC. (3) Studies with a total sample size of ≤20 patients. (4) Studies not reporting relevant data. (5) Non-comparative studies and review articles.

### Literature Search

Articles related to the review were searched by two reviewers independently. With the help of a librarian, the databases of PubMed and Embase were searched to identify relevant publications. All databases were screened from inception to 15th February 2021. We used the following keywords for the literature search: “ovarian cancer,” “lymph node,” “lymphadenectomy,” “pelvic,” “para-aortic,” “dissection,” “resection,” and “survival.” Supplementary Table 1 demonstrates the search strategy. Every search result was evaluated by the two reviewers independently, initially by their titles and abstracts and then by full texts of relevant publications. All full-texts were reviewed based on the inclusion and exclusion criteria and the article satisfying all the criteria was finally selected for this review. Any disagreements were resolved by discussion. To avoid any missed studies, the bibliography of included studies was hand searched for any additional references.

### Data Extraction and Risk of Bias Assessment

We prepared a data extraction form at the beginning of the review to extract relevant details from the studies. The final version of this template was approved by all the study investigators. Data of study authors, year of publication, study type, location, study groups and definition, sample size, age of the sample, histological type, percentage of metastasis detected in lymphadenectomy group, factors adjusted for multivariable analysis, and outcome data were extracted. Data were extracted by two reviewers independent of each other. Any disagreements were resolved by discussion.

The methodological quality of included studies was assessed using the Newcastle-Ottawa scale (28). This too was carried out in duplicate and independently by two study investigators. Studies were awarded points for selection of study population, comparability, and outcomes. The maximum score which can be awarded is nine.

### Statistical Analysis

We used “Review Manager” (RevMan, version 5.3; Nordic Cochrane Centre [Cochrane Collaboration], Copenhagen, Denmark; 2014) for the meta-analyses. Adjusted hazard ratios (HR) or related effect sizes of the outcomes were extracted along with the 95% confidence intervals (CI). Data were pooled using the generic inverse function of the meta-analysis software. Sub-group analysis was performed for the histological type of ESOCA random-effects model was preferred for the meta-analysis. The I^2^ statistic was used to assess inter-study heterogeneity. I^2^ values of 25–50% represented low, values of 50–75% medium, and more than 75% represented substantial heterogeneity. As <10 studies were included per meta-analysis, funnel plots were not used to assess publication bias. Random-effects meta-regression analysis was performed to assess the influence of the percentage of lymph node metastasis in the lymphadenectomy on the pooled effect size. Open MetaAnalyst software was used for the meta-regression analysis (29).

## Results

### Search Results and Details of Included Studies

**Figure 1:**
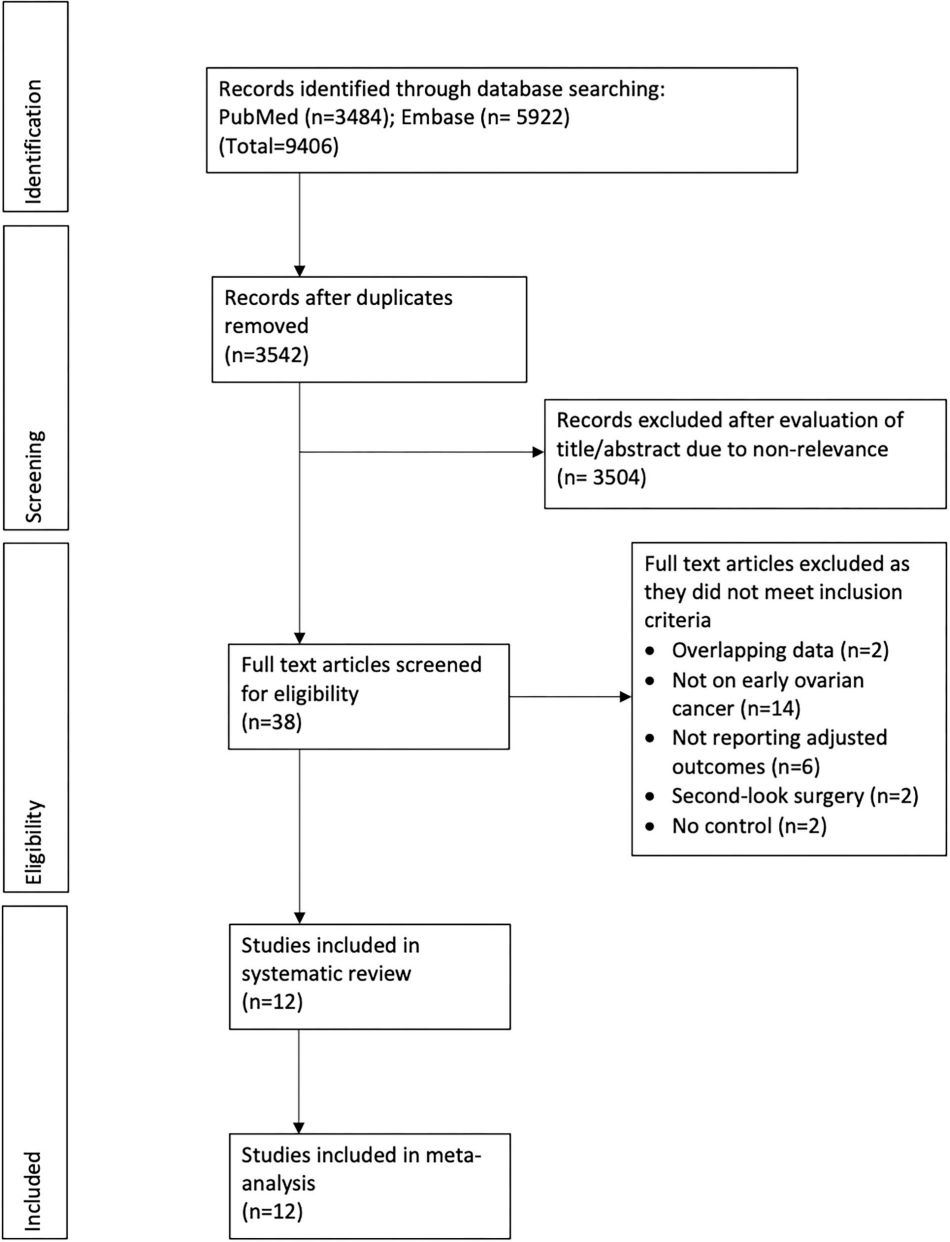
Study flow chart.

The flow-chart of the study is presented in Figure 1. Three thousand five hundred and forty two unique articles were identified after the literature search. After reviewing them by the titles and abstracts, we excluded 3,504 studies due to non-relevance with the review topic. Of the 38 studies selected for full-text analysis, 24 were excluded with reasons and a total of 12 studies were included in the review (30–41). Details of included studies are presented in Table 1. Only one study was an RCT while all others were retrospective observational studies. Six studies (30, 31, 33, 34, 36, 41) were conducted on epithelial tumors, three on malignant germ-cell tumors (32, 39, 40), two on sex cord-stromal tumors (35, 38) and one was only on ovarian carcinosarcoma (37). The sample size in the lymphadenectomy arm varied from 40 to 8,489 patients while in the control arm it varied from 22 to 4,628 patients. The number of patients with positive lymph nodes in the lymphadenectomy group varied from 0.8 to 25.8%. The number of lymph nodes removed in the lymphadenectomy group differed across studies. Similarly, there were variations in the factors adjusted in the multivariable analysis and the follow-up duration across studies.

**Table 1:** Details of included studies.

**References**	**Type**	**Location**	**Group**	**Definition**	**Sample size**	**Age (years)**	**Adjuvant chemotherapy (%)**	**Number of lymph nodes removed in LA group**	**Histological type**	**Factors adjusted in multivariable analysis**	**LN metastasis (%)**	**Follow-up**	**NOS score**
Maggioni et al. (30)	RCT	Italy	LA	Pelvic and para-aortic LA	138	51	56	Pelvic: 24 (15−33) Para-aortic:21 (15–30) Both: 47 (33–63)^∧^	Epithelial only	NR	22	87.8 months	9
			No-LA	Random sampling	130	52	66						
Abe et al. (31)	Observational	Japan	LA	Pelvic and/or para-aortic LA	40	56	97.3	Pelvic: 25 (9–79) Pelvic and para-aortic: 41 (21–80)^*^	Epithelial only	Residual tumor size, stage, histology, peritoneal cytology	6.9	31 months	7
			No-LA	Not performed	22	45	82.6						
Mahdi et al. (32)	Observational	USA	LA	Not defined	493	24	NR	11 (1–47)^*^	Germ cell only	Age, histology, race	10.5	60 months	6
			No-LA	Not performed	590	24.2							
Oshita et al. (33)	Observational	Japan	LA	Pelvic and para-aortic LA	284	53.5	87.3	34 {20–52}^#^	Epithelial only	Stage, histology, chemotherapy	8.1	65.8 months	7
			No-LA	Not performed	138	52	68.1						
Svolgaard et al. (34)	Observational	Denmark	LA	Pelvic/para-aortic LA or both	216	NR	NR	NR	Epithelial only	Cyst rupture, grade, histology, final stage, performance score, peritoneal cytology	6	38 months	6
			No-LA	Not performed	411	NR							
Nasioudis et al. (35)	Observational	USA	LA	Pelvic/para-aortic LA or both	572	50	NR	9 (1–61)^%^	Sex cord-stromal only	Age, stage, histology	3.3	95 months	6
			No-LA	Not performed	584	50							
Matsuo et al. (36)	Observational	USA	LA	>12 pelvic LA	8,489	NR	NR	11 [NR]^∧^	Epithelial only	Age, race, year of diagnosis, registry location, marital status, stage, histology, grade, tumor size	NR	7.1 years	6
			No-LA	<12 pelvic LA	4,628	NR							
Wang et al. (37)	Observational	USA	LA	Not defined	186	60.5	NR	NR	Carcinosarcoma only	Age, registry district, stage	25.8	NR	6
			No-LA	Not performed	177	65.4							
Erkilinç et al. (38)	Observational	Turkey	LA	Pelvic and para-aortic LA	47	54	NR	Pelvic: 15 (6–36) Para-aortic: 8 (8–34) ^%^	Sex cord-stromal only	Age, stage, number of mitosis	NR	48 months	7
			No-LA	Not performed	42	53							
Qin et al. (39)	Observational	China	LA	Not defined	126	25	83.3	NR	Germ cell only	Age, stage, histology, chemotherapy	0.8	68 months	7
			No-LA	Not performed	130	22.5	91.5						
Nasioudis et al. (40)	Observational	USA	LA	Not defined	1426	NR	56.2	9 (1–81) ^%^	Germ cell only	Age, insurance status, histology (dysgerminoma, non-dysgerminoma), presence of medical comorbidities and receipt of chemotherapy	10.3	62 months	6
			No-LA	Not performed	1348	NR	43.8						
Bizzarri et al. (41)	Observational	Italy	LA	Pelvic and para-aortic LA	360	54	100	32 (1–49) ^%^	Epithelial only	Age, serous histology, grade, disease stage	11.4	63 months	7
			No-LA	Not performed	129	60	100						

*LA, lymphadenectomy; NR, not reported; RCT, randomized controlled trial; LN, lymph node; Newcastle-Ottawa scale*.

∧ *Median {interquartile range}*.

* *Mean (Range)*.

# *Median {10–90 percentile}*.

% *Median (range)*.

### Meta-Analysis

**Figure 2:**
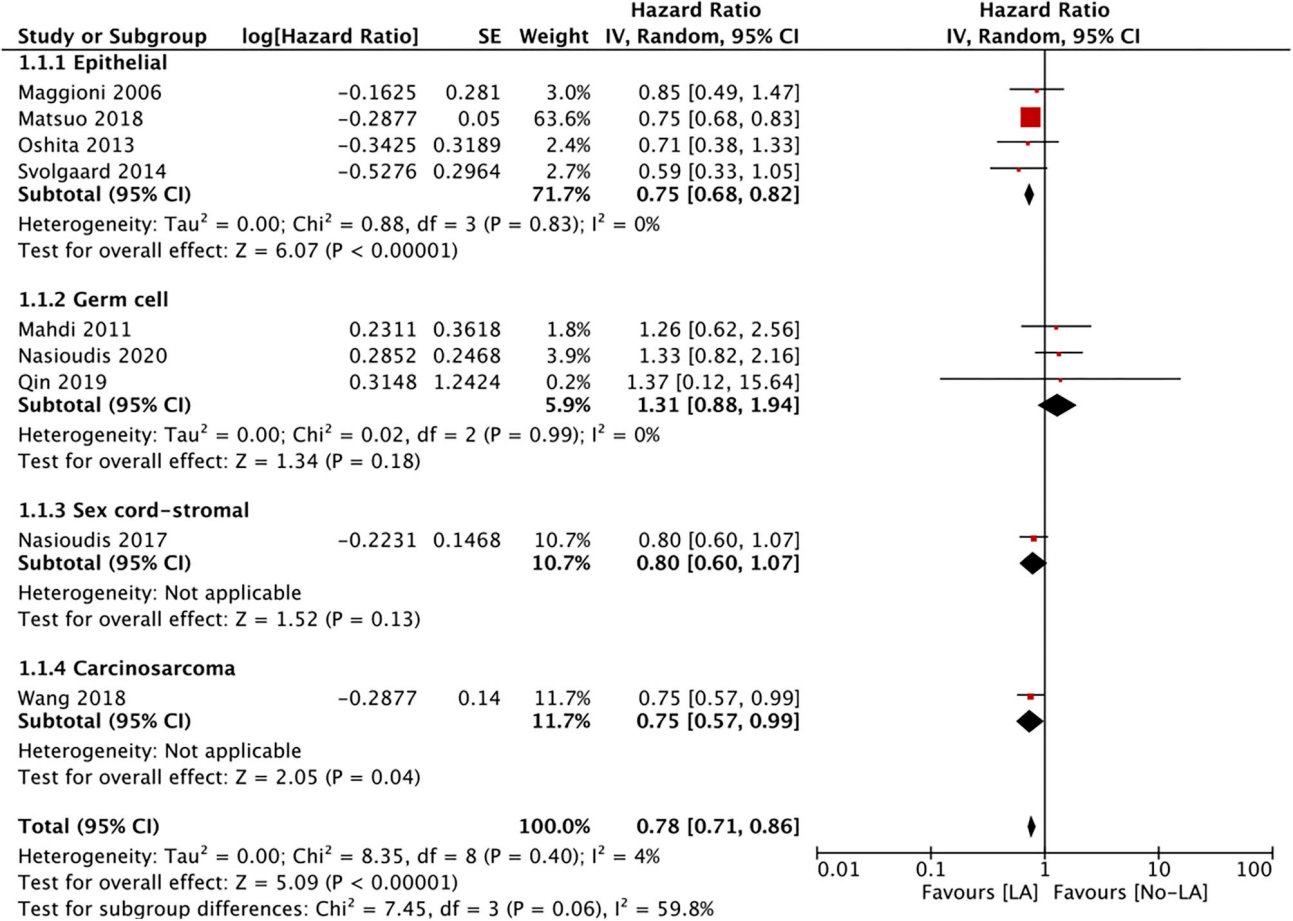
Meta-analysis of OS for ESOC between lymphadenectomy and control groups with sub-group analysis based on type of ESOC.

**Figure 3:**
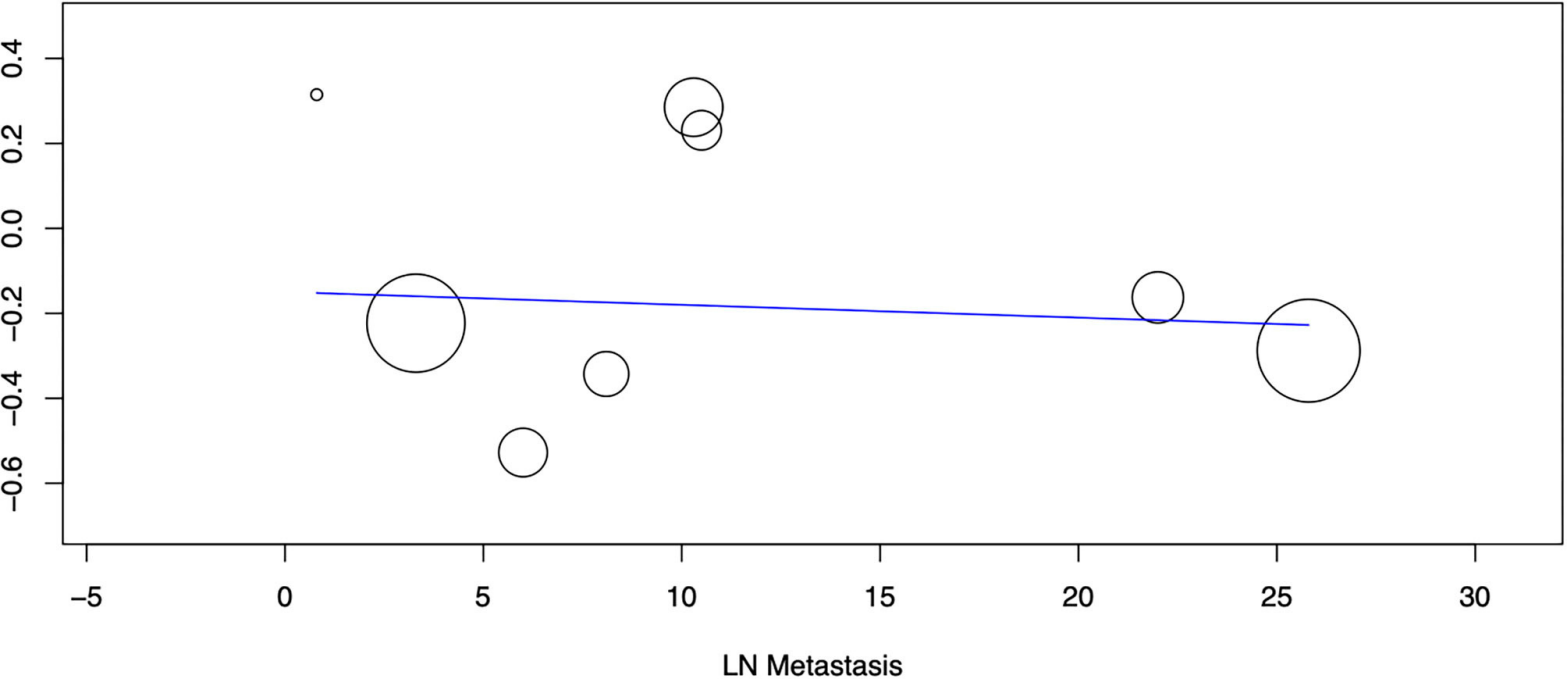
Meta-regression of the influence of percentage of lymph node metastasis (X-axis) on the pooled effect size of OS (Y-axis). Individual studies are represented by circles.

Nine studies reported data on OS. A meta-analysis of all studies irrespective of the histological type of ESOC indicated that lymphadenectomy is associated with significantly improved OS as compared to no lymphadenectomy (HR 0.78 95% CI 0.71, 0.86 I^2^ = 4% *p* < 0.00001) (Figure 2). On subgroup analysis, significant improvement in OS was noted only for epithelial tumors (HR 0.75 95% CI 0.68, 0.82 I^2^ = 0% p < 0.00001) but not for malignant germ cell tumors (HR 1.31 95% CI 0.88, 1.94 I^2^ = 0% *p* = 0.18). Analysis of single studies indicated a tendency of improved OS with lymphadenectomy which was significant for ovarian carcinosarcoma (HR 0.75 95% CI 0.57, 0.99 *p* = 0.04) but not for sex cord-stromal tumors (HR 0.80 95% CI 0.60, 1.07 *p* = 0.13) (Figure 2). On meta-regression, the percentage of patients with lymph node metastasis in the lymphadenectomy group was not found to influence the effect size (ß −0.003 95% CI −0.019, 0.013 *p* = 0.7) (Figure 3).

**Figure 4:**
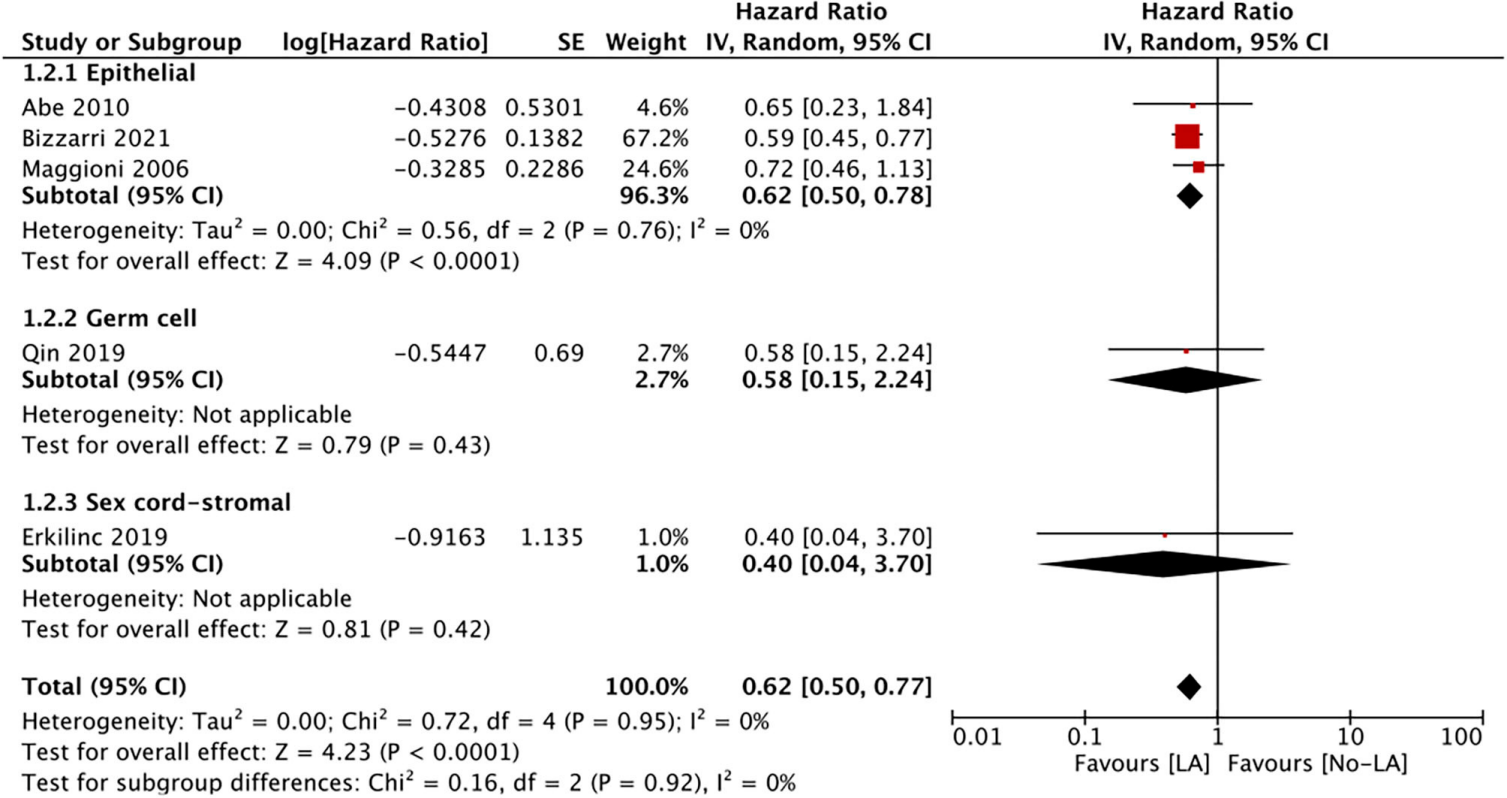
Meta-analysis of DFS for ESOC between lymphadenectomy and control groups with sub-group analysis based on type of ESOC.

Only five studies reported data on DFS. Meta-analysis of all studies irrespective of the histological type of ESOC indicated that lymphadenectomy is associated with significantly improved DFS as compared to no lymphadenectomy (HR 0.62 95% CI 0.50, 0.77 I^2^ = 0% *p* < 0.0001) (Figure 4). Data on epithelial tumors was reported by three studies and subgroup analysis indicated significantly improved DFS with lymphadenectomy (HR 0.62 95% CI 0.50, 0.78 I^2^ = 0% *p* < 0.0001). Single studies on malignant germ cell (HR 0.58 95% CI 0.15, 2.24 p = 0.43)and sex cord-stromal tumors (HR 0.40 95% CI 0.04, 3.70 *p* = 0.42) failed to demonstrate any significant beneficial effect of lymphadenectomy on DFS (Figure 4).

## Discussion

This systematic review and meta-analysis aimed to assess if lymphadenectomy improves OS and DFS in the case of ESOC. Our results, mainly from retrospective observation studies, indicate that lymphadenectomy significantly increases OS and DFS in ESOC; however, the results also depend on the tumor histology.

The recently conducted LION trial comparing lymphadenectomy vs. no-lymphadenectomy has demonstrated that lymphadenectomy has no significant beneficial effect on OS or DFS in cases of advanced ovarian cancer. On the contrary, serious complications like early mortality and repeat laparotomy were significantly increased in the lymphadenectomy group (42). Indeed, a significant limitation of retroperitoneal lymphadenectomy is the potential for several intraoperative and postoperative complications, like hemorrhage, sepsis, vascular injury, lymphocysts, intestinal or chylous fistula, lower limb edema, pulmonary embolism, repeat laparotomy and post-operative mortality (43). Therefore, it is important to clarify the role of this procedure especially in ESOC. To date, only one RCT has evaluated the role of lymphadenectomy for ESOC. Like the LION trial, Maggioni et al. (30) in their study too did not report any significant difference in OS and DFS between the study groups albeit with the overall effect size in favor of the lymphadenectomy group (OS, HR:0.85; DFS, HR:0.72). Indeed, high-quality rigorously conducted RCTs provide the best available evidence to guide clinical practice. However, when such evidence is scarce, pooled data from real-world observation studies is the second-best option. The RCT of Maggioni et al. (30) had its own set of limitations, like the small sample size and a low number of outcome events which hinder the applicability of their results. Data from our meta-analysis thus presents the best available evidence, to date, on the role of lymphadenectomy for ESOC. In contrast to the results of the lone RCT (30), our analysis demonstrated a significantly improved OS and DFS in ESOC patients undergoing lymphadenectomy as compared to the control group. The results, however, varied with the histological subtype.

Epithelial sub-type accounts for >85% of all cases of ovarian cancer (44). These tumors are also further classified into serous, endometrioid, clear cell, and mucinous carcinoma with differences in etiology, morphology, molecular biology, and prognosis (45). Due to limited data, we were unable to discern evidence on the role of lymphadenectomy in this subtype of epithelial ovarian cancer. All-inclusive, our analysis demonstrated a significantly improved OS as well as DFS with lymphadenectomy in cases of epithelial ESOC. This is in contrast with the past review of lymphadenectomy in ESOC by Chiyoda et al. (25) which reported improved OS but no difference in DFS. An increase in the sample size of the current analysis contributed to this difference. Another important point of consideration is the role of adjuvant chemotherapy, which is thought to improve outcomes for epithelial ESOC (46). However, many of the included studies lacked information on the use of adjuvant chemotherapy in the study groups. In the study of Maggioni et al. (30), there was bias in the use of chemotherapy as 90% lymph node-positive patients received chemotherapy compared to 56% node-negative patients. This may have contributed to the lack of difference in outcomes of the RCT. Oshita et al. (33) have shown that adjuvant chemotherapy may improve outcomes only in the non-lymphadenectomy group with no effect in patients with complete lymphadenectomy. Based on these results, it has been suggested that micro-metastasis in the lymph nodes in epithelial ESOC can be eliminated either via complete lymphadenectomy or adjuvant chemotherapy, and patients undergoing lymphadenectomy can avoid adjuvant chemotherapy (25, 33). Contrastingly, in the study of Bizzarri et al. (41) all patients in the lymphadenectomy and control group received adjuvant chemotherapy and lymphadenectomy was still associated with better DFS but not in OS as compared to no lymphadenectomy. Thus, given the significant heterogeneity in the studies for adjuvant therapy, strong conclusions cannot be derived and there is a need for large-scale RCTs to confirm the benefits of lymphadenectomy for epithelial ESOC.

Malignant ovarian germ cell tumors are a less common sub-type and are usually seen in a younger age group (47). These tumors have an excellent prognosis with >95% 5-year survival rates if the tumor is confined to the ovary (brown). Treatment guidelines for these tumors are extrapolated from epithelial sub-type and the role of lymphadenectomy is not very clear (40). Our analysis indicated that lymphadenectomy does not offer any survival advantage in malignant germ cell tumors. The difference in this outcome as compared to the epithelial sub-type can be attributed to the higher chemosensitivity of these tumors (48). Recent studies have demonstrated that postoperative chemotherapy is effective in managing these tumors with complete cure (49, 50). However, comprehensive staging is important in patients who cannot undergo adjuvant chemotherapy (49). In the absence of both, the significance of careful radiographic evaluation of lymph nodes, the need for re-operation for staging, and surveillance for grade 1 tumors have been suggested (40, 51). Given that these tumors affect the pediatric and adolescent age group, lesser invasive surgery would be beneficial and can avoid the complications associated with lymphadenectomy (47). However, our results should be interpreted with caution as only three studies were available for the OS analysis and only one study reported no difference in DFS with lymphadenectomy.

Similarly, limited data were available for sex-cord stromal tumors with only two studies; each indicating no difference in OS or DFS with lymphadenectomy. This is not surprising as these tumors are rare and similar to malignant germ cell tumors, their management is based on experience with epithelial tumors (47). The lack of difference in outcomes with lymphadenectomy can be due to the indolent nature of these tumors with rare lymph node metastasis as the disease is usually confined to the ovary (52). Lastly, ovarian carcinosarcoma is a rare variant of ovarian cancer also known as the mesodermal mixed tumor as it contains both epithelial and sarcomatous components (53). Optimal treatment is still not established but surgery and chemotherapy have shown good results (54). The tumor is known to be aggressive and associated with poor survival as compared to epithelial ovarian cancer (55). The lone study in an analysis indicated significantly better OS with lymphadenectomy with this tumor subtype.

The limitations of our review need to be specified. Foremost, except for epithelial tumors, data for other histological sub-types was limited. Several studies had to be excluded from our analysis as adjusted outcomes were not reported. Future studies should include reporting of adjusted HRs to allow clear delineation of outcomes. Secondly, the majority of the studies were retrospective in nature and would have been influenced by selection bias. An effort was made to minimize this by using only adjusted outcomes for the analysis. However, there was significant heterogeneity in the studies for outcomes adjusted with many not presenting data for adjuvant therapy. This may have skewed our results. Thirdly, the quality of lymphadenectomy could not be assessed and the number of lymph nodes removed was variable in the included studies. This is an important confounder that needs to be clarified in future studies. Fourthly, the definition of lymphadenectomy was not coherent across the included studies. Some studies performed both pelvic and para-aortic lymphadenectomy while in others only one technique was performed. Furthermore, some studies did not define lymphadenectomy *per se* in their cohorts. This could have been a major source of bias influencing the outcomes of this review. Lastly, an important component of any analysis on a surgical technique is its impact on complications. While we comprehensively discussed the role of lymphadenectomy on OS and DFS for ESOC, due to lack of data, no analysis on lymphadenectomy-related complications was possible. Future studies should report detailed data on the incidence of complications in order to better understand the role of lymphadenectomy for these tumors.

Nevertheless, the strengths include that our study is the first review focusing on all sub-types of ESOC. To minimize bias, only adjusted outcomes were pooled. A meta-regression was conducted to assess if the positivity of lymph nodes in the study group influences outcomes.

To conclude, within the limitations of the review, lymphadenectomy may improve OS and DFS for epithelial ESOC. Scarce data suggest that lymphadenectomy is not associated with improved outcomes for malignant germ cell and sex cord-stromal tumors but may benefit ovarian carcinosarcoma. Large-scale RCTs and robust observational studies shall improve current evidence.

## Data Availability Statement

The original contributions presented in the study are included in the article/Supplementary Material, further inquiries can be directed to the Corresponding author.

## Author Contributions

GY designed the study. Both the authors were involved in data acquisition, analysis, synthesis, wrote, edited, and approved the manuscript.

## Conflict of Interest

The authors declare that the research was conducted in the absence of any commercial or financial relationships that could be construed as a potential conflict of interest.
