# Supplementary material for: Impact of Lymphadenectomy on Outcomes of Early-Stage Ovarian Cancer: A Systematic Review and Meta-Analysis
Source: Front Surg. 2021 May 25;8:682348. doi: 10.3389/fsurg.2021.682348 (PMC8218907; doi:10.3389/fsurg.2021.682348)
Supplement: Supplementary file 1 [file Table_1.DOCX]

Supplementary Material

**Supplementary Table 1: Search strategy**

| **Query** | **Search Details** |
| --- | --- |
| **(((dissection) OR (resection)) AND (lymph node)) AND (ovarian cancer)** | **("dissect"[All Fields] OR "dissected"[All Fields] OR "dissecting"[All Fields] OR "dissection"[MeSH Terms] OR "dissection"[All Fields] OR "dissections"[All Fields] OR "dissects"[All Fields] OR ("resect"[All Fields] OR "resectability"[All Fields] OR "resectable"[All Fields] OR "resectates"[All Fields] OR "resected"[All Fields] OR "resecting"[All Fields] OR "resection"[All Fields] OR "resectional"[All Fields] OR "resectioned"[All Fields] OR "resectioning"[All Fields] OR "resections"[All Fields] OR "resective"[All Fields] OR "resects"[All Fields])) AND ("lymph nodes"[MeSH Terms] OR ("lymph"[All Fields] AND "nodes"[All Fields]) OR "lymph nodes"[All Fields] OR ("lymph"[All Fields] AND "node"[All Fields]) OR "lymph node"[All Fields]) AND ("ovarian neoplasms"[MeSH Terms] OR ("ovarian"[All Fields] AND "neoplasms"[All Fields]) OR "ovarian neoplasms"[All Fields] OR ("ovarian"[All Fields] AND "cancer"[All Fields]) OR "ovarian cancer"[All Fields])** |
| **((ovarian cancer) AND (pelvic)) AND (lymph node)** | **("ovarian neoplasms"[MeSH Terms] OR ("ovarian"[All Fields] AND "neoplasms"[All Fields]) OR "ovarian neoplasms"[All Fields] OR ("ovarian"[All Fields] AND "cancer"[All Fields]) OR "ovarian cancer"[All Fields]) AND ("pelvics"[All Fields] OR "pelvis"[MeSH Terms] OR "pelvis"[All Fields] OR "pelvic"[All Fields]) AND ("lymph nodes"[MeSH Terms] OR ("lymph"[All Fields] AND "nodes"[All Fields]) OR "lymph nodes"[All Fields] OR ("lymph"[All Fields] AND "node"[All Fields]) OR "lymph node"[All Fields])** |
| **((ovarian cancer) AND (para-aortic)) AND (lymph node)** | **("ovarian neoplasms"[MeSH Terms] OR ("ovarian"[All Fields] AND "neoplasms"[All Fields]) OR "ovarian neoplasms"[All Fields] OR ("ovarian"[All Fields] AND "cancer"[All Fields]) OR "ovarian cancer"[All Fields]) AND "para-aortic"[All Fields] AND ("lymph nodes"[MeSH Terms] OR ("lymph"[All Fields] AND "nodes"[All Fields]) OR "lymph nodes"[All Fields] OR ("lymph"[All Fields] AND "node"[All Fields]) OR "lymph node"[All Fields])** |
| **((ovarian cancer) AND (lymphadenectomy)) AND (survival)** | **("ovarian neoplasms"[MeSH Terms] OR ("ovarian"[All Fields] AND "neoplasms"[All Fields]) OR "ovarian neoplasms"[All Fields] OR ("ovarian"[All Fields] AND "cancer"[All Fields]) OR "ovarian cancer"[All Fields]) AND ("lymph node excision"[MeSH Terms] OR ("lymph"[All Fields] AND "node"[All Fields] AND "excision"[All Fields]) OR "lymph node excision"[All Fields] OR "lymphadenectomies"[All Fields] OR "lymphadenectomy"[All Fields]) AND ("mortality"[MeSH Subheading] OR "mortality"[All Fields] OR "survival"[All Fields] OR "survival"[MeSH Terms] OR "survivability"[All Fields] OR "survivable"[All Fields] OR "survivals"[All Fields] OR "survive"[All Fields] OR "survived"[All Fields] OR "survives"[All Fields] OR "surviving"[All Fields])** |
